# Supplementary material for: Evaluation of Hi-C Sequencing for Detection of Gene Fusions in Hematologic and Solid Tumor Pediatric Cancer Samples
Source: Cancers (Basel). 2024 Aug 23;16(17):2936. doi: 10.3390/cancers16172936 (PMC11394547; doi:10.3390/cancers16172936)
Supplement: Supplementary file 1 [file cancers-16-02936-s001.zip › Figure S1.pdf]

Supplementary Materials:

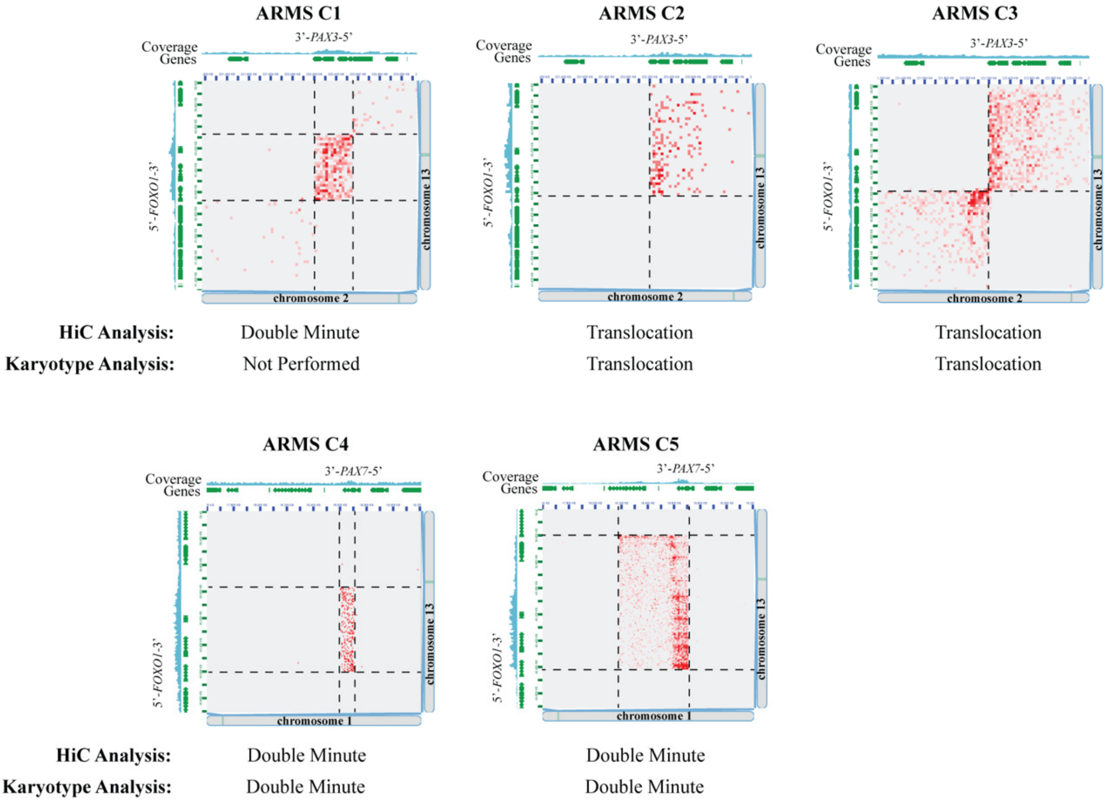

Figure S1. HiC analysis determines the rearrangement type from formalin-fixed paraffin-embedded solid tumors and is concordant with karyotype analysis. The HiC signal pattern around the breakpoint calls inform the type of rearrangement. For simple inter-chromosomal translocations (translocations without co-occurring events at the breakpoint, such as inversions or deletions), there is a single breakpoint and the pattern of HiC signal strength will be strongest between pairs of loci directly flanking the breakpoints on each chromosome, and will dissipate for pairs of loci moving further away from the breakpoint. For simple reciprocal translocations, this looks like a bowtie pattern when viewing the loci around the breakpoint in a HiC heatmap, with strongest HiC signal strength at the center of the bowtie and with HiC signal strength dissipating bilaterally. For double minutes formed between genomic regions derived from two different chromosomes, the HiC signal pattern appears as a (often small) rectangle, with strongest HiC signal at opposing corners of the rectangle, and the HiC signal largely contained between the pair of genomic regions on the double minute. The Figure shows HiC heatmaps from the five alveolar rhabdomyosarcoma (ARMS) cases, zoomed-in to the loci surrounding the clinically significant gene fusion call. Genomic coordinates, gene locations, and sequencing coverage from each chromosome are shown along the edges of the X and Y axes of the heatmaps. The gene positions and orientations of the genes involved in the gene fusion are indicated. Black dashed lines depict the breakpoint location(s) on each chromosome. Cases "ARMS C1", "ARMS C4", and "ARMS C5" show the expected pair of breakpoints and HiC signal pattern of double minutes, whereas cases "ARMS C2" and "ARMS C3" show the expected HiC signal pattern of inter-chromosomal translocations. In all Hi-C heatmaps, pairs of loci with more Hi-C read support appear as darker red entries in the Hi-C heatmap, pairs of loci with less Hi-C read sup.
